# Genital VZV in a Third Trimester Pregnancy and the Critical Role of Interdisciplinary Planning

**DOI:** 10.1155/2024/1937661

**Published:** 2024-04-12

**Authors:** Brenna Cook, Caroline Shadowen, Lorna Clark, Alena Hoover, Stephanie Lee, Whitney Bender

**Affiliations:** ^1^School of Medicine, Virginia Commonwealth University, Richmond, VA, USA; ^2^Department of Obstetrics and Gynecology, Virginia Commonwealth University, Richmond, VA, USA; ^3^Department of Maternal Fetal Medicine, Virginia Commonwealth University, Richmond, VA, USA

## Abstract

**Introduction:**

Herpes simplex (HSV) and varicella zoster (VZV) viruses are harmful infectious agents in pregnancy due to their ability to impact maternal-fetal dyads through various modalities including vertical transmission, neonatal infection, and maternal morbidity. As a result, accurate diagnosis and prompt treatment of these infections in pregnancy is critical.

**Case:**

A 19-year-old primigravida presented to our tertiary care center at 30 weeks' gestation with vulvar swelling, burning, and pain. Workup included direct PCR testing of a particularly erythematous area of the vulva which returned positive for VZV. The patient was treated with a 10-day course of acyclovir with resolution of her symptoms. She later had a full-term spontaneous vaginal delivery outside of the infectious window with no significant morbidity for either her or her neonate.

**Conclusion:**

Although a rare presentation, the presence of a genital lesion or labial swelling during pregnancy warrants workup for VZV, particularly among patients known to be varicella nonimmune. If genital VZV is diagnosed during pregnancy, the development of contingency plans through interdisciplinary collaboration should be pursued to ensure a safe delivery and postpartum course for both the maternal-fetal dyad as well as other patients on the unit and the provider care team.

## 1. Introduction

Herpes simplex viruses 1 and 2 (HSV-1 and -2) and varicella zoster virus (VZV) belong to the herpesvirus family and classically cause primary infections characterized by lesions of skin and mucosal surfaces [1]. HSV and VZV are particularly important in pregnancy due to their ability to infect the fetus or neonate through placental transmission, ascending infection of the amniotic fluid, or via physical contact with lesions during or after delivery [2]. While HSV is classically associated with transmission through contact with genital lesions during delivery leading to neonatal HSV infections, VZV can cause either fetal or neonatal disease depending upon the time of maternal infection and fetal exposure. VZV in pregnancy is also associated with a risk of maternal complications, most commonly pneumonia, which occurs in 20% of pregnant women with varicella and carries a mortality rate of up to 15%.

Primary maternal VZV infections that occur in the first 20 weeks of pregnancy can lead to congenital varicella syndrome (CVS), characterized by fetal limb hypoplasia, microcephaly, cicatricial skin scarring, chorioretinitis, and long-term neurodevelopmental delays [3]. While CVS occurs in less than 1% of primary maternal varicella infections, it carries a poor prognosis with up to 30% mortality in the first several weeks of life [3]. In contrast to CVS, primary maternal varicella infections occurring at later gestations can result in neonatal varicella, characterized by fever and vesicular rash, with the potential for progression to varicella pneumonia, hepatitis, or meningoencephalitis. Primary maternal varicella infections that occur in the final four weeks of pregnancy lead to neonatal varicella in 23% of infants [4]. Transmission during this period most often occurs through ascending infection or direct contact during or after delivery.

Herpes zoster (HZ), which occurs as a result of latent VZV reactivation from peripheral ganglia, is characterized by a painful, vesicular rash that occurs in a dermatomal distribution [1]. In contrast to primary varicella, HZ infections during pregnancy have not been associated with the same fetal risks. This is likely due to maternal transmission of VZV antibodies to the fetus, which are protective against in utero infection. In addition, because most cases of HZ occur in cervical and thoracic dermatomes, resulting in lesions on the head, trunk, and extremities, transmission through direct contact with the fetus during or after delivery is uncommon [5]. In one case series of over 300 cases of maternal HZ during pregnancy, no cases of CVS were reported [3].

In addition to maternal and neonatal morbidity from primary varicella infection, virus-naïve patients, clinical care team members, and other contacts are at risk of infection due to the highly contagious nature of the VZV. Varicella carries up to a 90% infection rate in nonimmune individuals. While many women of childbearing age are immune to varicella from either historical primary infection or prior vaccination, up to 2% of pregnant patients are not immune and are, therefore, at high risk of infection during the vulnerable antenatal period [6].

Because the virus can harm the mother and fetus/neonate as well as infect others, accurate diagnosis and prompt treatment of maternal VZV infection are critical. We present a case that reinforces the importance of including VZV in the differential diagnosis of a pregnant patient presenting with vulvar symptoms and highlights the role of collaborative interdisciplinary care in the management of antenatal VZV to best ensure safety for both the affected maternal-fetal dyad as well as other patients and team members.

## 2. Case

A 19-year-old primigravida presented to the Labor and Delivery (L&D) triage unit at our tertiary medical center at 30 weeks gestation reporting two days of vulvar swelling, burning, and pain. These symptoms were preceded by several days of rhinorrhea, myalgias, and malaise. Prior to presentation, her pregnancy course was notable only for chlamydia treated at eight weeks of pregnancy (with negative test of cure in the third trimester) and sickle cell trait. A fetal anatomic ultrasound survey at 20 weeks gestation was unremarkable. She had previously had VZV vaccination (2004 and 2007) and had no history of chickenpox per patient report. Her VZV IgG serology on initial prenatal labs, however, was negative, suggesting the nonimmune status.

**Figure 1:**
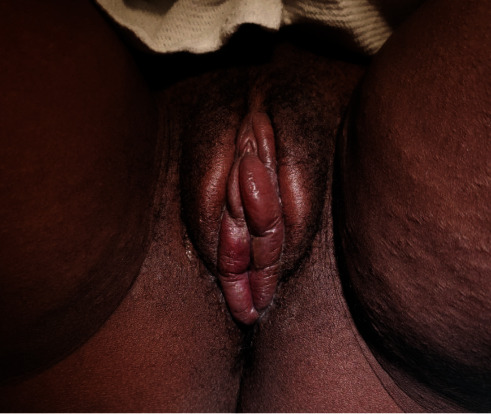
Initial presenting exam in the triage unit of L&D. Specimen obtained from left labia majus.

On arrival to our triage unit, the patient was afebrile with normal vital signs. Physical exam revealed significant edema, erythema, and tenderness to palpation of the labia majora, minora, and vestibule, with physiologic discharge from the vagina (Figure 1). No discrete lesions were noted, but there was a small area of increased erythema on the innermost aspect of the left labia majus from which a specimen for HSV/VZV direct PCR testing was obtained. No additional lesions were noted. The patient had reassuring fetal monitoring and was discharged home with recommendations for symptomatic treatment including oral antihistamines and ice packs.

Direct testing returned positive for VZV and negative for HSV. Laboratory methodology used at our institution to differentiate VZV and HSV includes a single PCR test with distinct analytes for each virus. Although her negative VZV Ig serology in early pregnancy raised suspicion for primary VZV infection, the localized and unilateral nature of her symptoms was more consistent with genital HZ. As a result, the differential diagnosis included both unusual presentation of primary VZV and genital HZ.

The patient was treated with oral acyclovir 800 milligrams five times per day for 10 days. She was advised to self-isolate at home for seven days or until the lesions crusted over. She had a telephone consultation with maternal fetal medicine to review the potential fetal and maternal risks of infection in pregnancy. Given her late gestational age at presentation, she was counseled on the low likelihood of congenital varicella syndrome. A diagnostic amniocentesis was discussed; this procedure was not completed after shared decision-making. A growth scan after clinical recovery was recommended to screen for fetal growth restriction. We discussed that her risk of neonatal varicella syndrome would be dependent upon gestational age at delivery; specifically, we reviewed that in the absence of preterm delivery, she was highly unlikely to have an affected neonate. Lastly, we discussed the maternal complications that can arise from varicella infection in pregnancy. The patient was counseled to present for care in the event of intractable fever or new respiratory symptoms.

Follow-up exam three weeks later confirmed complete resolution of her symptoms. A growth ultrasound at 36 weeks was normal (estimated fetal weight 2407 grams, 17^th^ percentile). The remainder of her pregnancy was uncomplicated, and the patient had a term spontaneous vaginal delivery. Serial newborn exams were normal with no evidence of prenatal or neonatal infection. The mother and infant were discharged two days after delivery, and postpartum follow-up has been unremarkable.

## 3. Discussion

Our case reinforces the importance of considering VZV when evaluating patients with genital lesions or labial swelling during pregnancy. To our knowledge, localized genital varicella has been reported 15 times in case reports (only one occurred in an immunocompetent pregnant female) [5, 7–15]. In the previously documented pregnancy case, genital HZ was diagnosed at near-term in a known VZV-immune patient. Our case was initially less clear due to our patient's VZV nonimmune status.

Historically, genital lesions in VZV have been documented in 2% of the cases. However, infections involving the genitals may be more prevalent than previously thought. A study analyzing samples from over two thousand adults with skin and mucosal lesions found VZV in 6% of the patients. Ten percent of VZV-positive samples in this study were obtained from genital sites [4]. This further highlights the importance of considering VZV when evaluating genital lesions during pregnancy. In addition, while maternal HZ has not shown to carry the same fetal risks as those associated with primary varicella, the risks associated with genital HZ at or near term are lesser known given the rarity of this presentation.

Our case also highlighted the importance of using both laboratory data and clinical judgement when distinguishing primary varicella from HZ. From a laboratory standpoint, our patient had a previously negative VZV antibody test on routine prenatal labs and subsequent direct testing that was positive for VZV, which supported a diagnosis of primary varicella. From a clinical perspective, the localized and unilateral nature of her symptoms pointed towards a diagnosis of genital HZ. Upon further investigation of the laboratory techniques used at our institution, we found that while this patient's VZV antibody level did not meet the threshold for a positive (immune) test result (0.8 antibody index), the antibody level was 0.2, indicating the presence of a small amount of VZV antibodies in the patient's blood. This combination of laboratory findings and clinical presentation supports a diagnosis of HZ resulting from the reactivation of VZV from the sacral ganglia.

**Figure 2:**
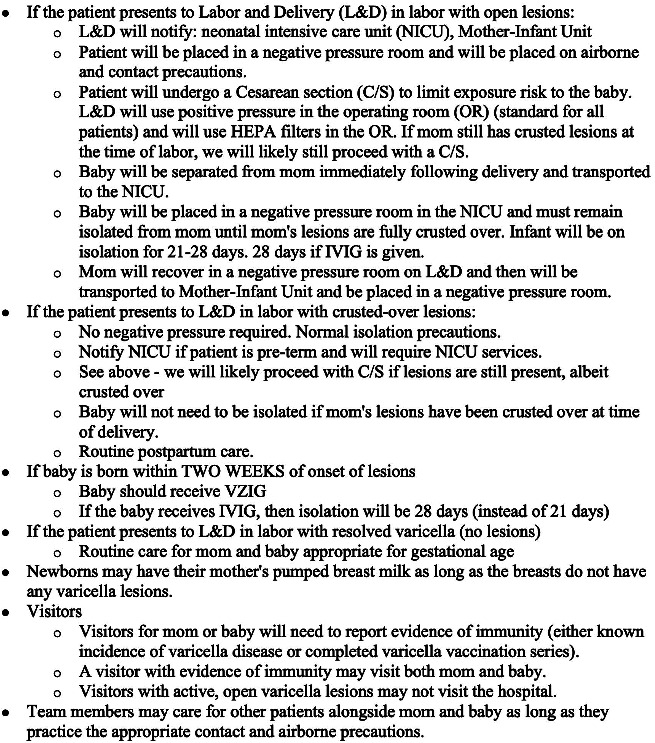
Contingency plans developed by the multidisciplinary team.

Our case also highlighted the need for multidisciplinary planning to ensure a safe delivery and postnatal course for the patient, her neonate, and her contacts. In the case of our patient, a meeting was held with neonatology, newborn nursery, pediatrics, infectious disease, epidemiology, maternal fetal medicine, and L&D leadership. This collaborative work led to the development of contingency plans for each of the following scenarios: if the patient presented to L&D in labor with open lesions; if the patient presented to L&D in labor with crusted lesions; if the baby was born within two weeks of onset of lesions; and if the patient presented to L&D with resolution of lesions. The visitor policy for potentially exposed family and friends in each of these timepoints was also discussed. These outlined plans were communicated to a representative of each specialty team (Figure 2).

While our patient's course fortunately concluded in an uncomplicated term delivery four weeks after the resolution of her lesions, this interdisciplinary collaboration ensured a detailed plan of care should her situation have been different. The protocols developed aimed to minimize risk of our patient's infection transmitting to other pregnant women and their neonates and outlined a clear role for each subspecialty in each situation. While protocols exist to guide medical management of VZV during pregnancy, there are not currently national guidelines that include the role of interdisciplinary care in managing peripartum VZV infections [16, 17]. L&D units should consider developing standardized guidelines regarding VZV management that address the scenarios outlined above.

## Data Availability

The data used to support the findings of this study are available as part of the article and no other additional source data are required.
